# Outbreak of Exanthematous Illness Associated with Zika, Chikungunya, and Dengue Viruses, Salvador, Brazil

**DOI:** 10.3201/eid2112.151167

**Published:** 2015-12

**Authors:** Cristiane W. Cardoso, Igor A.D. Paploski, Mariana Kikuti, Moreno S. Rodrigues, Monaise M.O. Silva, Gubio S. Campos, Silvia I. Sardi, Uriel Kitron, Mitermayer G. Reis, Guilherme S. Ribeiro

**Affiliations:** Secretaria Municipal de Saúde de Salvador, Salvador, Brazil (C.W. Cardoso);; Fundação Oswaldo Cruz, Salvador (I.A.D. Paploski, M. Kikuti, M.S. Rodrigues, M.M.O. Silva, U. Kitron, M.G. Reis, G.S. Ribeiro);; Universidade Federal da Bahia, Salvador (I.A.D. Paploski, M. Kikuti, G.S. Campos, S.I. Sardi, M.G. Reis, G.S. Ribeiro);; Emory University, Atlanta, Georgia, USA (U. Kitron)

**Keywords:** Zika virus, chikungunya virus, dengue virus, flavivirus, arboviruses, viruses, disease outbreak, epidemiology, acute exanthematous illness, vectorborne infections, Salvador, Brazil

**To the Editor:** Zika virus (ZIKV) has been recognized as an emerging mosquito-borne flavivirus since outbreaks were reported from Yap Island in 2007 (*1*), French Polynesia in 2013 (*2*), and Cook Island and New Caledonia in 2014 (*3*). It has joined dengue virus (DENV) and chikungunya virus (CHIKV) as global public health threats (*4*). ZIKV infection typically causes a self-limited dengue-like illness characterized by exanthema, low-grade fever, conjunctivitis, and arthralgia, and an increase in rates of Guillain-Barré syndrome have been observed during ZIKV outbreaks (*5*).

In Brazil, clusters of cases of acute exanthematous illness have been reported from various regions since late 2014, and in April 2015, ZIKV was identified as the etiologic agent (*6*). In May 2015, the Brazilian Ministry of Health recognized circulation of ZIKV in Brazil. We report epidemiologic findings for an ongoing outbreak of acute exanthematous illness in the population of Salvador, the third largest city in Brazil.

The Salvador Epidemiologic Surveillance Office (ESO) was first alerted to cases of an acute exanthematous illness early in 2015. Reporting of cases increased during March, and in April the ESO established 10 public emergency health centers in Salvador as sentinel units for systematic surveillance of patients with acute exanthematous illness of unknown cause. The units searched retrospectively for suspected cases by review of medical charts of patients treated since February 15, continued with prospective case detection, and submitted weekly reports of identified cases to the ESO.

During February 15−June 25, a total of 14,835 cases of an indeterminate acute exanthematous illness were reported from the 12 sanitary districts in Salvador. The overall attack rate was 5.5 cases/1,000 persons (4.6 cases/1,000 men and 6.3 cases/1,000 women, 8.2 cases/1,000 children <15 years of age, 5.4 cases/1,000 persons 15–39 years of age, and 3.8 cases/1,000 adults >40 years of age).

The epidemic curve peaked in the first week of May, which was 1 week after molecular diagnosis of ZIKV in 8 patients residing ≈50 km from Salvador and during a period of intense media coverage of the outbreak (Figure) (*6*). Reporting of suspected dengue cases in Salvador did not vary substantially from that in other years and was >5 times lower: 2,630 cases, of which 165/366 (45.1%) were positive for dengue IgM, 20/590 (3.4%) positive for dengue virus nonstructural protein 1, and 1/11 (9.1%) positive for dengue virus by reverse transcription PCR (Figure). During the same period, 58 cases of suspected chikungunya were reported and 24 patients with suspected Guillain-Barré syndrome were hospitalized.

**Figure:**
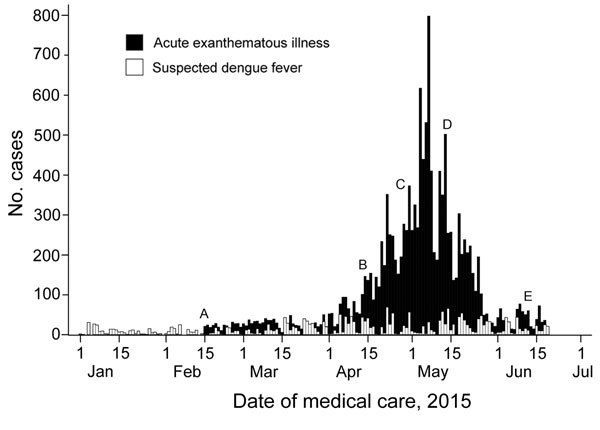
Reported cases of indeterminate acute exanthematous illness and suspected dengue fever in Salvador, Brazil, by date of medical care, February 15−June 25, 2015. Letters indicate specific events. A) February 15: systematic reporting of cases of acute exanthematous illness of unknown cause begins in Salvador. B) April 13: Salvador Epidemiologic Surveillance Office releases its first epidemiologic alert about the outbreak in Salvador. C) April 29: Zika virus is confirmed in 8 samples from patients residing ≈50 km from Salvador (http://portalsaude.saude.gov.br/index.php/situacao-epidemiologica-dados-dengue-2) and media coverage of the outbreak intensifies (http://www.correio24horas.com.br/detalhe/noticia/doenca-misteriosa-que-atinge-cidades-baianas-e-identificada-como-zika-virus/?cHash = 74792c41f3128395ba0ffa5e1ed9dbbe). D) May 14: Brazilian Ministry of Health announces circulation of Zika virus in Brazil (http://portalsaude.saude.gov.br/index.php/o-ministerio/principal/secretarias/svs/noticias-svs/17702-confirmacao-do-zika-virus-no-brasil). E) June 11: Brazilian press announces that cases of Zika virus infection have been confirmed in 8 states in Brazil (http://www1.folha.uol.com.br/cotidiano/2015/06/1640752-virus-primo-da-dengue-zika-ja-tem-casos-confirmados-em-oito-estados.shtml).

The median age of case-patients was 26 years (interquartile range 11–39 years), but all age groups were affected, which is a pattern typical of spread of new microorganisms (or subtypes) in a susceptible population. Median duration of symptoms at time of medical attention was 1 day (interquartile range 0–3 days). All patients had exanthema and most (12,711/14,093 [90.2%]) had pruritus. Fever (4,841/13,786, 35.1%), arthralgia (278/1,048 [26.5%]), headache (3,446/13,503 [25.6%]), and myalgia (223/1,033 [21.6%]) were less common.

Serum samples from some patients were examined for rubella IgM (2/200, 1.0% positive), rubella IgG (15/18, 83.3% positive), measles IgM (0/11, 0% positive), dengue nonstructural protein 1 (3/185, 1.6% positive), dengue IgM (17/80, 21.3% positive), parvovirus B19 IgM (0/1, 0% positive), and parvovirus B19 IgG (1/1, 100% positive). Reverse transcription PCR was performed on 58 serum samples stored at −20°C and confirmed ZIKV in 3 (5.2%) samples, CHIKV in 3 (5.2%) samples, DENV type 3 in 1 (1.7%) sample, and DENV type 4 in 1 (1.7%) sample.

Identification of ZIKV, CHIKV and DENV as etiologic agents of acute exanthematous illness suggests that these 3 *Aedes* spp. mosquito−transmitted viruses were co-circulating in Salvador and highlights the challenge in clinically differentiating these infections during outbreaks. Although we were not able to determine the specific incidence of each virus, the low frequency of fever and arthralgia, which are indicators of dengue and chikungunya, point to ZIKV as the probable cause of several of the reported cases. Furthermore, laboratory-confirmed cases of infection with ZIKV were simultaneously identified in other cities within metropolitan Salvador (*6**,**7*) and in other states in Brazil (*8*). Low diagnosis of ZIKV infection is likely because viremia levels among infected patients appear to be low (*9*).

The spread of ZIKV represents an additional challenge for public health systems, particularly because of the risk for concurrent transmission of DENV and CHIKV by the same vectors, *Ae. aegypti* and *Ae. albopictus* mosquitoes, which are abundant throughout tropical and subtropical regions. To date, the largest outbreak of chikungunya in Brazil occurred in 2014 in Feira de Santana, Bahia, ≈100 km from Salvador, where dengue is also prevalent (*10*).

This report illustrates the potential for explosive simultaneous outbreaks of ZIKV, CHIKV, and DENV in the Western Hemisphere and the increasing public health effects of *Aedes* spp. mosquitoes as vectors. The apparent increase in reports of Guillain-Barré syndrome during the outbreak deserves further investigation to elucidate whether this syndrome is associated with ZIKV infection. Public health authorities in Brazil and neighboring countries should plan accordingly.

## References

[1] Duffy MR, Chen T-H, Hancock WT, Powers AM, Kool JL, Lanciotti RS, Zika virus outbreak on Yap Island, Federated States of Micronesia. N Engl J Med. 2009;360:2536–43. 10.1056/NEJMoa0805715 19516034

[2] Cao-Lormeau VM, Roche C, Teissier A, Robin E, Berry AL, Mallet HP, Zika virus, French Polynesia, South Pacific, 2013. Emerg Infect Dis. 2014;20:1085–6. 10.3201/eid2011.141380 24856001 PMC4036769

[3] Roth A, Mercier A, Lepers C, Hoy D, Duituturaga S, Benyon E, Concurrent outbreaks of dengue, chikungunya and Zika virus infections—an unprecedented epidemic wave of mosquito-borne viruses in the Pacific, 2012–2014. Euro Surveill. 2014;19:20929 . 10.2807/1560-7917.ES2014.19.41.20929 25345518

[4] Musso D, Cao-Lormeau VM, Gubler DJ. Zika virus: following the path of dengue and chikungunya? Lancet. 2015;386:243–4. 10.1016/S0140-6736(15)61273-9 26194519

[5] Musso D, Nilles EJ, Cao-Lormeau V-M. Rapid spread of emerging Zika virus in the Pacific area. Clin Microbiol Infect. 2014;20:O595–6. 10.1111/1469-0691.12707 24909208

[6] Campos GS, Bandeira AC, Sardi SI. Zika virus outbreak, Bahia, Brazil. Emerg Infect Dis. 2015;21:1885–6. 10.3201/eid2110.150847 26401719 PMC4593454

[7] Zammarchi L, Tappe D, Fortuna C, Remoli ME, Günther S, Venturi G, Zika virus infection in a traveller returning to Europe from Brazil, March 2015. Euro Surveill. 2015;20:21153 . 10.2807/1560-7917.ES2015.20.23.21153 26084316

[8] Zanluca C, De Melo VC, Mosimann AL, Dos Santos GI, Dos Santos CN, Luz K. First report of autochthonous transmission of Zika virus in Brazil. Mem Inst Oswaldo Cruz. 2015;110:569–72 . 10.1590/0074-02760150192 26061233 PMC4501423

[9] Lanciotti RS, Kosoy OL, Laven JJ, Velez JO, Lambert AJ, Johnson AJ, Genetic and serologic properties of Zika virus associated with an epidemic, Yap State, Micronesia, 2007. Emerg Infect Dis. 2008;14:1232–9 . 10.3201/eid1408.080287 18680646 PMC2600394

[10] Teixeira MG, Andrade AM, Costa MC, Castro JN, Oliveira FL, Goes CS, East/Central/South African genotype chikungunya virus, Brazil, 2014. Emerg Infect Dis. 2015;21:906–7 . 10.3201/eid2105.141727 25898939 PMC4412231

